# Capture of isoflurane from anaesthetised dogs and cats following methadone, medetomidine and ketamine administration

**DOI:** 10.1002/vetr.70212

**Published:** 2025-12-26

**Authors:** Florence Hillen, David Yates, Kate White

**Affiliations:** ^1^ School of Veterinary Medicine and Science University of Nottingham Nottingham UK; ^2^ Bolton RSPCA Bolton UK

## Abstract

**Background:**

Volatile anaesthetic agents contribute to climate change, and reducing this impact is a commendable ambition. We evaluated the efficiency of a veterinary capture device for isoflurane (VET‐can/VET‐dock, SageTech Veterinary) in anaesthetised cats and dogs.

**Methods:**

Purposeful sampling was used to recruit enough animals undergoing isoflurane general anaesthesia to fill one VET‐can to at least 95% capacity. The vaporiser and VET‐can were weighed pre‐ and post‐anaesthesia to determine in vivo mass transfer (MT) of isoflurane and water. The primary outcomes were capture efficiency (isoflurane delivered: desorbed, %) and in vivo MT. The secondary outcomes were associations between MT and patient characteristics and anaesthesia variables.

**Results:**

Thirty anaesthetics (21 cats and nine dogs) were required to fill the VET‐can to capacity. The overall capture efficiency was 81% isoflurane with 4% water' as it's the isoflurane capture efficiency that matters here and the water capture explains the difference in overall efficiency and MT.

**Limitations:**

Interchangeable use of sidestream and mainstream capnography resulted in a difference in times to end‐tidal isoflurane 0%.

**Conclusions:**

Capture efficiency was 81%. The device prevented a mean release of 3.2 kgCO_2_e for each 20‐minute anaesthetic period, approximately the equivalent of 13 miles driven in an average UK car.

## INTRODUCTION

Climate change threatens global health^1^ and survival, and consequently, it is both a social responsibility and a business imperative that the carbon footprint of all activities is evaluated. There are limited data describing the veterinary healthcare footprint, but there is concern and recognition of the importance of reducing contamination.^2^, ^3^ A veterinary practice's gas emissions can be classified into three scopes. Scope 1 covers direct emissions from the practice: burning fuel, company vehicles, greenhouse gases (GHG) from air conditioning, refrigeration and release of gaseous anaesthesia into the environment. Scope 2 covers indirect emissions from the purchase and use of electricity, heating and cooling by the practice. The practice's use of energy is indirectly responsible for the release of these GHG emissions. Scope 3 includes all other indirect emissions that occur in the upstream and downstream activities of the practice (e.g., consumables, drugs and associated services, business travel, employee and client commuting, waste disposal, use of sold products, transportation, and both up‐ and downstream distributions). Scope 3 emissions are believed to account for 70%‒90% of most companies’ carbon footprint.^4^

Accurate measurement of these emissions is challenging but addressing them is fundamental for a credible climate strategy and will facilitate prioritising interventions with the greatest and most efficient impact first on the basis of these quantitative analyses. The tool for healthcare emissions accounting is lifecycle assessment, an internationally standardised (ISO 14040) method for estimating environmental impacts, including from resource extraction, manufacturing, packaging, transport, use and waste disposal. The anaesthesia journals have published both primary research and editorials on anaesthesia's role in GHG emissions,^2^, ^3^, ^5^, ^6^, ^7^, ^8^, ^9^, ^10^, ^11^ and the environmental impact of different surgical procedures has been evaluated.^12^, ^13^ It is possible to estimate a carbon footprint from direct and indirect GHGs of an individual product, operation or the sector as a whole. Carbon dioxide is responsible for 80%–85% of the global warming potential and is considered the major GHG. Other GHGs, such as methane, nitrous oxide, chlorofluorocarbons and volatile anaesthetic agents, can be considered and are reported as carbon dioxide equivalents (CO_2_e). This aggregate metric is the carbon footprint. Carbon hotspots that have been identified include medical devices, consumables, transport and electricity.^12^, ^13^ There is general agreement across the sector on reducing single‐use devices, avoiding N^2^O, and adopting low‐flow anaesthesia, but there is still a long way to go in implementing these interventions. The carbon emissions for a canine tibial plateau levelling osteotomies have recently been estimated as 63.4 kgCO_2_e, with 21% resulting from volatile anaesthesia.^14^

To prevent the release of harmful GHGs in the form of waste anaesthetic gases, it is possible to insert a volatile capture device (VCD) downstream of the anaesthetic machine by connecting it to the scavenging port.^15^ The optimal VCD would offer an integrated, low‐emission system that could adsorb the anaesthetic vapour for subsequent recycling. Companies manufacturing the VCD technology (Baxter, Blue Zone Technologies and SageTech Medical) claim in vitro mass transfer (MT) rates of 99%‒100% on their websites, although these claims describe capture once the volatile agent is trapped in the cannister, as opposed to medical capture efficiencies, which describe the total mass of volatile agent extracted from canisters available to repurpose and for which rates of 25%‒51% have been reported.^15^, ^16^ The SageTech technology uses activated carbon and has been tested in vitro with sevoflurane and desflurane^17^, ^18^ and clinically in humans with sevoflurane, isoflurane^19^ and desflurane.^16^ A 65% capture efficiency and 77% in vivo MT of isoflurane from dogs and cats undergoing anaesthesia have recently been described.^20^

The aim of the current study was to assess in vivo isoflurane MT and capture efficiency in cats and dogs under anaesthesia following methadone, medetomidine and ketamine administration.

## METHODS

The study was designed as a prospective controlled clinical study at the RSPCA Bolton Branch. Reporting follows the ARRIVE 2.0 and STROBE guidelines.

### Volatile agent capture

Purposeful sampling was used to enrol enough cases to fill one cannister (VET‐can) to 500 g, or more than 99% fill, to ensure efficient desorption. The veterinary VCD (VET‐can/VET‐dock, SageTech) consisted of a VET‐can (in its VET‐dock) connected between the scavenging port and the active scavenging system and leakage of volatile agent is prevented by two self‐sealing valves. This was achieved using corrugated tubing to connect the exhaust port of the breathing system to the Vet‐dock and the outlet of the Vet‐dock to the passive scavenging arrangement. The VET‐can was removed from the VET‐dock between collection days. The scavenging port of the anaesthetic monitor (Datex‐Ohmeda S/5, GE Healthcare) was also connected to the inlet of the VET‐dock. A single vaporiser was filled at the start of each day, and both the vaporiser and VET‐can were weighed before and after each anaesthetic using a precision balance to within 0.1 g (CGOLDEN‐WALL). For each anaesthetic, the in vivo MT was calculated as the difference between VET‐can weight change (pre‒post procedure) divided by the vaporiser weight change (pre‒post procedure). At the end of the study, the VET‐can was returned to the manufacturer for isoflurane extraction from the adsorbent. The capture efficiency was calculated as the overall mass of volatile anaesthetic desorbed from the single VET‐can used as a percentage of mass delivered.

### Animals

The inclusion criteria were healthy adult cats or dogs (ASA 1‒2) requiring anaesthesia for neutering or surgery within the charity's scope and provision of care. Food, but not water, was withheld overnight prior to anaesthesia. Preoperatively, patients were examined, weighed and assigned a body condition score.

### Anaesthesia

The breathing systems and anaesthetic machines were leak‐tested prior to anaesthesia each day. All dogs received 0.2 mg/kg methadone (Comfortan; Dechra) and 0.02 mg/kg medetomidine (Sedator; Dechra) intramuscularly (im), followed 10 minutes later by 2 mg/kg ketamine (Anesketin; Dechra) im. All cats received 6 mg/m^2^ methadone (0.33‒0.50 mg/kg), 600 µg/m^2^ (33‒50 µg/kg) medetomidine and 60 mg/m^2^ (3.3‒5.0 mg/kg) ketamine im. The trachea was intubated with an appropriately sized cuffed PVC tube and inflated to a cuff pressure of 25‒30 cmH_2_O (AG Cuffill; Hospitech Respiration). Anaesthesia was maintained with isoflurane (Isofane; Covetrus) in 100% oxygen delivered via a modified paediatric Mapleson D (Infant T‐piece) for patients weighing less than 10 kg or a circle breathing system for patients weighing 10 kg or more. The animals were positioned on a heated operating table and continuously monitored with pulse oximetry, oscillometric non‐invasive blood pressure (PetMap graphic II, PetMap), capnography and agent end‐tidal concentrations (Datex S/5; GE Healthcare, or Capnotrue MG, Bluepoint Medical). All dogs received 0.2 mg/kg meloxicam subcutaneously (sc) prior to surgery, and cats received 0.3 mg/kg.

On completion of surgery, the vaporiser was turned off, and the time recorded until the end‐tidal isoflurane concentration (${\mathrm{ET}}_{\mathrm{ISO}}^{\prime }$) measured 0% using either mainstream or sidestream capnography, depending on equipment availability. Thereafter, the patient was disconnected from the breathing system, which was immediately capped to continue residual capture from the tubing for an additional 2 minutes. Post‐procedure, 0.1 mg/kg sc atipamezole (Atipam, Dechra) was administered to cats (as part of a concurrent study). Following each procedure, anaesthetic data were used to calculate a hypotensive index and minimum alveolar concentration (MAC) hours (Table 1).

**TABLE 1:** Calculation of the hypotensive index and minimum alveolar concentration (MAC).

Value	Definition	Calculation
Hypotensive index 60	Duration over which mean arterial blood pressure is less than 60 mmHg	Sum over monitored time intervals of (60 ‒ mean blood pressure) × the time in hours that the mean blood pressure was less than 60 mmHg
MAC‐hours	Duration of exposure to the volatile anaesthetic agent over time	(Average end‐expired volatile agent concentration/MAC volatile agent) × hours of volatile agent anaesthesia^a^

^a^ An isoflurane MAC of 1.36 vol% (dogs) and 1.69 vol% (cats) was used.^21^

### Study outcomes

The primary outcomes were the capture efficiency of the VET‐can and in vivo MT. The secondary outcomes were associations between in vivo MT and patient‐ and anaesthesia‐related factors. The carbon savings from avoiding atmospheric release of isoflurane were also estimated using a specific gravity of 1.50 at 20°C and a carbon conversion of isoflurane of 0.87 kgCO_2_e/mL.^14^, ^22^

### Statistical analysis

Patient characteristics, in vivo MT and anaesthesia data are reported as median (range). Univariate exploratory analysis was performed using Spearman correlations between in vivo MT and patient‐ and anaesthesia‐related variables (GraphPad Prism version 10.4.1).

## RESULTS

Thirty animals (21 cats and nine dogs) were recruited for the study on seven non‐consecutive days between January and March 2025 (Tables 2 and 3). Four cats required additional isoflurane via a tight‐fitting mask for induction of anaesthesia. Two of these cats did not receive the full dose of medetomidine, methadone and ketamine. Uncuffed tubes (4.5 mm) were used in six patients. One cat was euthanased at the end of surgery following owner consent after a diagnosis of feline immunodeficiency virus.

**TABLE 2:** Summary of patient characteristics and procedural data for 30 animals’ anaesthetics where volatile agent capture was performed.

	Results
Procedure	8 castrates 18 ovariectomies 1 ovariohysterectomy 2 wound flushes 1 mammary strip
Breed (canine)	Chihuahua (two females) French Bulldog (three females) Jack Russell Terrier (one female) Labrador Retriever (one female) Staffordshire Bull Terrier (one male) Yorkshire Terrier (one male)
Breed (feline)	Bombay (one female) Domestic Shorthair (seven males, 11 females) Ragdoll (one female) Unknown breed (one female)
Weight, median (range) (kg)	
Feline	3.3 (1.75‒6.0)
Canine	9.4 (2.1‒28.4)
Body condition score, median (range) (1: underweight; 9: obese)	4 (3‒7)
Age, median (range) (years)	1 (0.4‒9.5)

**TABLE 3:** Anaesthetic and capture data for the 30 patients.

Variable	Number of anaesthetics	Value
In vivo MT (%) all animals	30	86 (57‒100)
In vivo MT (%) circle breathing system	2	91.5 (91‒92)
In vivo MT (%) modified paediatric Mapleson D (Infant T‐piece)	19	86 (57‒100)
Length of anaesthesia (minutes)	30	75 (15‒123)
${\mathrm{ET}}_{\mathrm{ISO}}^{\prime }$ during maintenance of anaesthesia (%)	30	1.1 (0.8‒1.6)
Time from ceasing isoflurane delivery to ${\mathrm{FI}}_{\mathrm{ISO}}^{\prime }$ of 0% (side stream analyser) (seconds)^a^	19	368 (106‒810)
Time from ceasing isoflurane delivery to ${\mathrm{FI}}_{\mathrm{ISO}}^{\prime }$ of 0% (mainstream analyser) (seconds)	10	82 (17‒222)
Hypotensive (60) index	30	0 (0‒5.33)
Mean arterial blood pressure (mmHg)	30	109 (78‒144)
${\mathrm{PE}}_{CO_{2}}^{\prime }$ (mmHg)	28	46 (34‒59)
SpO_2_ (%)	27	98 (94‒99)
MAC‐multiples	27	0.7 (0.5‒1.1)
MAC‐hours of anaesthesia	27	0.8 (0.2‒1.4)

*Note*: Values are expressed as median (range). A hypotensive index was calculated for each anaesthetic as the sum over monitored time intervals of (60 ‒ mean blood pressure) × time in hours that the mean blood pressure was less than 60. MAC‐multiples were calculated as end‐tidal volatile agent divided by isoflurane MAC of 1.36 vol% (dogs) and 1.69 vol% (cats).^21^ MAC‐hour was calculated as (average end‐expired volatile agent concentration/MAC volatile agent) × (hours of volatile agent anaesthesia) based on an isoflurane MAC of 1.36 vol% (dogs) and 1.69 vol% (cats).^21^

Abbreviations: MAC, minimum alveolar concentration; MT, mass transfer.

^a^ One male cat was euthanased at the end of the procedure after the diagnosis of feline immunodeficiency virus.

### Primary outcome

The manufacturer extracted 527 g of liquid from the VET‐can (a measured gain in VET‐can weight on the precision scales of 538.3 g). This represented a 105% fill rate. The overall water captured was 21 g or 4%. The weight of isoflurane extracted from the VET‐can was 506 g. The weight of change of the vaporiser due to isoflurane use totalled 626.7 g. This resulted in an isoflurane capture efficiency of 81% for the whole cohort.

The median in vivo MT (or percentage capture by weight) across 30 anaesthetics was 86% (57%‒100%). Assuming a consistent water vapour content of 4%, the capture efficiency of these 30 anaesthetics would be 82%.

In all anaesthetics, an ${\mathrm{ET}}_{\mathrm{ISO}}^{\prime }$ of 0% was reached prior to disconnection of the breathing system and extubation. One cat was euthanased at the end of the procedure.

### Secondary outcomes

Spearman correlations revealed the following relationships between in vivo MT and weight (cats) (*r*_s_ = 0.23, *p* = 0.32), weight (dogs) (*r*_s_ = 0.8, *p* = 0.33), body condition score (*r*_s_ = 0.1, *p* = 0.66), age (*r*_s_ = ‒0.19, *p* = 0.38), length of capture (time until ${\mathrm{ET}}_{\mathrm{ISO}}^{\prime }$ 0%) (*r*_s_ = 0.013, *p* = 0.95), MAC‐multiples (*r*_s_ = 0.15, *p* = 0.21), MAC‐hours (*r*_s_ = 0.09, *p* = 0.66), mean arterial blood pressure (*r*_s_ = ‒0.33, *p* = 0.077), end‐tidal carbon dioxide concentration ($ET_{CO_{2}}$) (*r*_s_ = 0.21, *p* = 0.29) and SpO_2_ (%) (*r*_s_ = 0.14, *p* = 0.48).

## DISCUSSION

The overall capture efficiency of 81% isoflurane in this study is higher than 25%‒51% capture efficiencies reported in medical literature and 65% in the only published veterinary study.^20^ The in vivo MT ranged from 57% to 100%, with a median of 86%, which is similar to that reported by White et al. Water vapour capture rates (4%) were similar to reports (5.4%‒8.6%) using sevoflurane and isoflurane^23^, ^24^, ^25^ but significantly less than human desflurane studies (31%). However, different VCDs and incompletely filled capture devices may have been responsible for these differences.^17^ Irrespective of the capture ambitions, lower flow anaesthesia is widely recognised as a priority in national guidelines^26^ and reduces emissions in a modelled small animal study (targeting 1 L/min) by 63%.^27^ In human healthcare, studies have shown that strategically placed algorithms with visual reminders about flow rate and volatile choice can dramatically reduce volatile use and offer significant cost savings to the organisation and benefit the environment.^28^ One in vitro study also found that MT was higher at lower fresh gas flow (FGF) and vaporiser settings.^17^

In our study, the non‐rebreathing Mapleson D (Infant T‐piece) was used in all but two anaesthetics, where a circle was chosen due to the patient weights (24 and 28 kg). For feline patients less than 4 kg, the FGFs were not reduced below 2 L/min in this observational study in order to maintain the clinical routine. The demographic of UK dogs is predisposed to smaller breeds,^29^ and for anaesthetising small patients, veterinary surgeons often rely on non‐rebreathing systems, which intrinsically require higher FGFs. Transitioning to increased use of paediatric circle systems is a key option for volatile saving and in addition is likely to improve the efficiency of capture technology, although this can be limited by lack of training or suitable circle systems. Additionally, scrutinising the FGF used for non‐rebreathing systems and using capnography to ensure that the FGF is appropriate is key in carbon accounting. This clinic has a high feline caseload and neuters more than 1000 cats per year and, to date, had favoured the use of the Ayres T‐piece for ease of use, cost, disposal (in the case of feline respiratory disease) and familiarity. In this study, the anaesthetic protocol (medetomidine‒methadone‒ketamine‒isoflurane) prevented rapid emergence or necessity for sudden extubation in recovery, in contrast to propofol or alfaxalone protocols, ensuring that the capture of volatile agent could be maximised before the administration of atipamezole and extubation. One study demonstrated that two‐thirds of the volatile agent capture occurred during washout,^30^ and it can take 71 hours to eliminate 99.9% isoflurane from the brain after 3 hours of isoflurane anaesthesia,^31^ since considerable volatile presence in the tissues remains at extubation. As an increase in capture efficiency occurs during the washout phase, it stands to reason to continue to scavenge until the endotracheal tube is removed to maximise capture and reduce workplace contamination.

Our study and others^20^, ^30^ found no association between in vivo MT and MAC‐hours or duration of anaesthesia. A study in people found correlation between lower in vivo MT and high end‐tidal agent percentage at the time of extubation and longer anaesthetics (4 hours).^16^ All animals in the present study reached 0% isoflurane at ET_disconnect_, but in view of the MT range (57%‒100%), it supports the observation that an ${\mathrm{ET}}_{\mathrm{ISO}}^{\prime }$ of 0% does not guarantee complete elimination of isoflurane from the animal. Multimodal analgesia reduces the MAC of isoflurane, and it may have been possible to reduce further the volatile agent delivered by infiltrating local anaesthetic for castrates and wound repairs and utilising locoregional approaches such as the transverse abdominus plane block^32^ for female neuters, all of which were performed via a ventral midline approach.

It is highly likely that the recovery of the volatile agent from the tissues is dependent on several factors, such as cardiac output, ventilation, body fat percentage^33^ and the solubility of the agent in the tissues.^34^ White and colleagues demonstrated a correlation between hypotension and lower in vivo MT in one population of dogs and cats, albeit with a different anaesthesia induction protocol to the current study. Hypotension was uncommon in the current study; median mean arterial pressure (MAP) was 109 mmHg (78‒144 mmHg), and it was therefore not possible to draw conclusions about in vivo MT and hypotension. Isoflurane has a higher blood gas solubility compared to the other volatile agent with market authorisation in small animals (sevoflurane) meaning more volatile agent accumulates in muscle, splanchnic organs, and especially fat, which acts as an isoflurane reservoir up until the anaesthetic partial pressure in the tissue equilibrates with that in arterial blood. Once isoflurane administration is discontinued, anaesthetic released from these reservoirs redistributes to the vessel‐rich tissue group including the brain and spinal cord and opposes elimination from these sites of action. The consequence is greater context sensitivity on the isoflurane elimination half‐life.^33^ In theory, it is possible that in vivo MT will be reduced in obese patients for this reason; however, in morbidly obese humans, the effect on recovery is negated somewhat by the reduced blood flow to adipose tissue with increasing body fat percentage and the long‐time constants, making the problem clinically insignificant.^35^ The effect of obesity on in vivo MT and efficiency of capture has yet to be investigated. Lifecycle analyses describe the total carbon impacts of a product or system, including all potential savings but also the costs. The process emissions of this veterinary VCD are being undertaken but are not yet published. An independent lifecycle analysis for the supplier's human medical VCD in a 10‐theatre human hospital suggested that with a 65% capture efficiency, the process emissions represent 3.1% of the avoided atmospheric emissions (SageTech data, available on request). The reprocessed isoflurane has yet to gain market authorisation, but the ambition of moving from a linear (take‒make‒waste) to a circular (reuse‒repair‒recycle) economy is a commendable ambition. Atmospheric release of isoflurane captured during the 1705 minutes of anaesthesia could have produced 293 kgCO_2_e. Volatile agent capture avoided release of 0.17 kgCO_2_e/min of anaesthesia or 3.4 kgCO_2_e for each 20‐minute anaesthetic period, which approximates to the equivalent of 14 miles driven in an average UK car.

## LIMITATIONS

This study had a small number of canine patients, which was limited by the season (winter/early spring) and the high requirement for feline neuters. During the study, two multiparameter monitors were utilised because of a concurrent study at another site, and the second device used for the final 10 patients had mainstream carbon dioxide and agent concentration rather than side stream. This difference will have likely resulted in closer to real‐time ET_ISO_ percentage in the final 10 patients as the gas sample is not aspirated into the machine for analysis.^36^, ^37^ Likewise, it was observed that the drop of fractional inspired isoflurane was instantaneous in the mainstream monitor and times to ET_ISO_ 0% were more rapid.^37^ Associations between patient characteristics and in vivo MT were explored as secondary outcome measures, meaning that an insufficient number of animals may have been included to detect relationships, and future studies are warranted.

## CONCLUSIONS

We demonstrated capture efficiencies in dogs and cats higher than those previously reported in people and animals, with easy integration into a busy charity practice operating theatre. The capture efficiencies demonstrate that VCDs are a carbon saving system that can complement existing reduction strategies such as flow rate optimisation and circle system use. Altruistically, it could also be imagined that incorporating VCDs can act as reminders and prompt us to consider other green interventions in both theatre and the rest of the practice environment.

## AUTHOR CONTRIBUTIONS

*Contributed to the idea, hypothesis and study design*: Kate White and Florence Hillen. *Contributed to data acquisition*: Kate White, Florence Hillen and David Yates. *Assembled the data*: Kate White and Florence Hillen. *Performed the statistical analysis*: Kate White and Florence Hillen. *Interpreted the data and prepared the first draft*: all authors. *Commented on, edited, reviewed and finally approved the last version of the correspondence*: all authors.

## CONFLICT OF INTEREST STATEMENT

The University of Nottingham provided additional monitoring. SageTech Veterinary provided a VET‐can and VET‐dock and the precision scales at no cost. The authors retained full control of the study design, implementation of data collection and interpretation. The authors declare they have no conflicts of interest.

## ETHICS STATEMENT

Ethical approval was granted from the University of Nottingham, School of Veterinary Medicine and Science ethics committee (3924230901).

## Data Availability

The data that support the findings of this study are available from the corresponding author upon reasonable request.
